# Safety and efficacy of laparoscopic digestive tract nutrition reconstruction combined with conversion therapy for patients with unresectable and obstructive gastric cancer

**DOI:** 10.3389/fonc.2023.1175580

**Published:** 2023-06-08

**Authors:** Rong Ye, Chuandong Wang, Bo Hu, Guoxian Guan

**Affiliations:** ^1^ Department of Colorectal Surgery, the First Affiliated Hospital of Fujian Medical University, Fuzhou, China; ^2^ Department of Colorectal Surgery, National Regional Medical Center, Binhai Campus of the First Affiliated Hospital, Fujian Medical University, Fuzhou, China; ^3^ Shengli Clinical Medical College of Fujian Medical University, Fuzhou, China; ^4^ Department of Gastrointestinal Surgery, Xiamen Humanity Hospital Fujian Medical University, Xiamen, China; ^5^ Fujian Abdominal Surgery Research Institute, the First Affiliated Hospital, Fujian Medical University, Fuzhou, China

**Keywords:** advanced gastric cancer, digestive tract reconstruction, conversion therapy, immune and nutrition status, inflammatory status

## Abstract

**Background:**

To explore the safety, efficacy, and survival benefits of laparoscopic digestive tract nutrition reconstruction (LDTNR) combined with conversion therapy in patients with unresectable gastric cancer with obstruction.

**Methods:**

The clinical data of patients with unresectable gastric cancer with obstruction who was treated in Fujian Provincial Hospital from January 2016 to December 2019, were analyzed. LDTNR was performed according to the type and degree of obstruction. All patients received the epirubicin + oxaliplatin + capecitabine regimen as conversion therapy.

**Results:**

Thirty-seven patients with unresectable obstructive gastric cancer underwent LDTNR, while thirty-three patients received chemotherapy only. In LDTNR group patients, the proportion of nutritional risks gradually decreased, the rate of severe malnutrition decreased, the proportion of neutrophil-lymphocyte ratio (NLR) <2.5 increased, the proportion of prognosis nutrition index (PNI) ≥45 increased, and the Spitzer QOL Index significantly increased at day 7 and 1 month postoperatively (P<0.05). One patient (6.3%) developed grade III anastomotic leakage and was discharged after the endoscopic intervention. The median chemotherapy cycle of patients in LDTNR group was 6 cycles (2-10 cycles), higher than that in Non-LDTNR group (P<0.001). Among those who received LDTNR therapy, 2 patients had a complete response, 17 had a partial response, 8 had stable disease, and 10 had progressive disease, which was significantly better than the response rate in Non-LDTNR group(P<0.001). The 1-year cumulative survival rates of the patients with or without LDTNR were 59.5% and 9.1%. The 3-year cumulative survival rate with or without LDTNR was 29.7% and 0%, respectively (P<0.001).

**Conclusions:**

LDTNR can improve the inflammatory and immune status, increase compliance with chemotherapy, and have potential benefits in improving the safety and effectiveness of and survival after conversion treatment.

## 1 Introduction

Gastric cancer is frequently diagnosed at an unresectable advanced stage and has a poor prognosis (1). Gastric obstruction (GO), a common complication in such patients, has various symptoms, such as nausea and vomiting, depriving them of further anticancer treatments (2, 3). A previous study demonstrated that fewer chemotherapy cycles and an objective response rate (ORR) could be achieved in patients with gastric outlet obstruction (GOO) (4). Therefore, ameliorating GO plays an important role in the continuation of subsequent anticancer treatments.

The National Comprehensive Cancer Network (NCCN) guidelines recommend the restoration of enteral nutrition with palliative methods, including gastrojejunostomy (GJ), gastrostomy (GT), and jejunostomy (JT) (5). Moreover, with the application of laparoscopic techniques, these palliative methods tend to afford quicker resumption of enteral nutrition, less surgical trauma, and better compliance with chemotherapy (6). Our previous study also indicated that laparoscopic gastrojejunostomy (LGJ) combined with conversion therapy could improve the overall survival (OS) of these patients (4). However, few studies have focused on multimodal therapy of laparoscopic digestive tract nutrition reconstruction (LDTNR), including GJ, GT, and JT, combined with conversion therapy. Therefore, the present study was designed to determine the safety and efficacy of LDTNR combined with conversion therapy in patients with GO. The results of this study may aid in clinical decisions to enable the management of symptoms caused by GO.

## 2 Methods

### 2.1 Patients

A retrospective study was conducted on all cases of unresectable gastric cancer treated at the Fujian Provincial Hospital, Fuzhou, Fujian, China, between January 2016 and December 2019. The inclusion criteria were as follows (1): pathological and radiological diagnosis of gastric cancer and presence of non-curable factors (No.16 lymph node metastasis, peritoneum metastasis and other organ invasion or metastasis) (7); (2) GO confirmed by endoscopy; (3) difficulty in oral intake caused by GO; (4) tolerance to general anesthesia and laparoscopic surgery; and (5) written informed consent. Exclusion criteria were as follows: (1) combined with other gastrointestinal obstructions; (2) combined with other malignant tumors; (3) patient received other anticancer treatments, such as chemoradiotherapy, before surgery; (4) severe dysfunction of the heart, lungs, kidneys, and other important organs; (5) patient completed less than two chemotherapy cycles; and (6) incomplete clinicopathological data. Based on the inclusion criteria, 70 patients were enrolled in the study. A multidisciplinary team determined the strategy for each patient. Gastric outlet obstruction scoring system (GOOSS) was scored as followed: 0 = no oral intake, 1 = liquids only, 2 = soft food and 3 = solid food. Patients with a GOOSS score of 2 were categorized into the Non-LDTNR group, and GOOSS score of 0 or 1 were categorized into the LDTNR group. The study was approved by the Ethics Committee of the Fujian Provincial Hospital. All procedures were performed in accordance with the Declaration of Helsinki of 1964 and its later versions.

### 2.2 Surgical procedures

All patients in LDTNR group received general anesthesia with endotracheal intubation and were placed in a supine position with splayed legs. We used the “five-hole method” to establish the laparoscopic hole. In the “five-hole method,” a 10 mm Trocar was inserted 2 cm below the umbilicus as the observation hole. A 12 mm Trocar was inserted 2 cm below the costal margin of the left anterior axillary line as the main operating hole. Trocars of 5 mm were inserted 2 cm above the plain umbilical of the left midclavicular line and 2 cm below the costal margin of the right anterior axillary line as an auxiliary operation hole. And a 5 mm Trocar was inserted 2 cm above the right midclavicular plain umbilicus.

**Figure 1:**
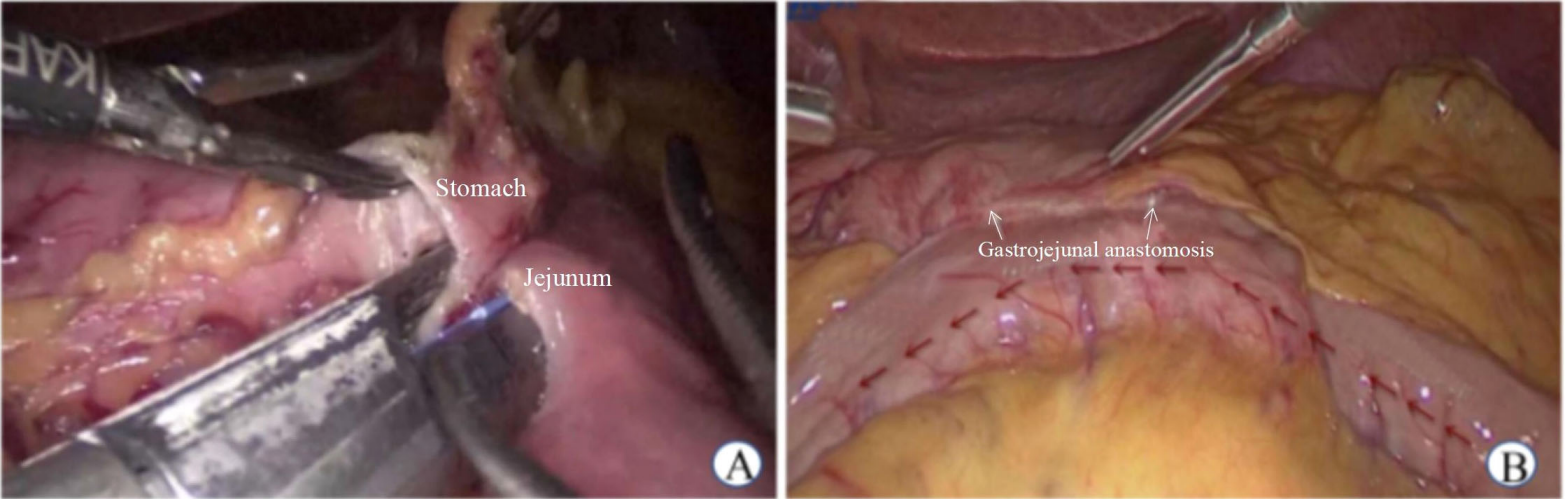
Laparoscopic gastrojejunostomy. **(A)** Side to side gastrojejunostomy **(B)** Postoperative status.

**Figure 2:**
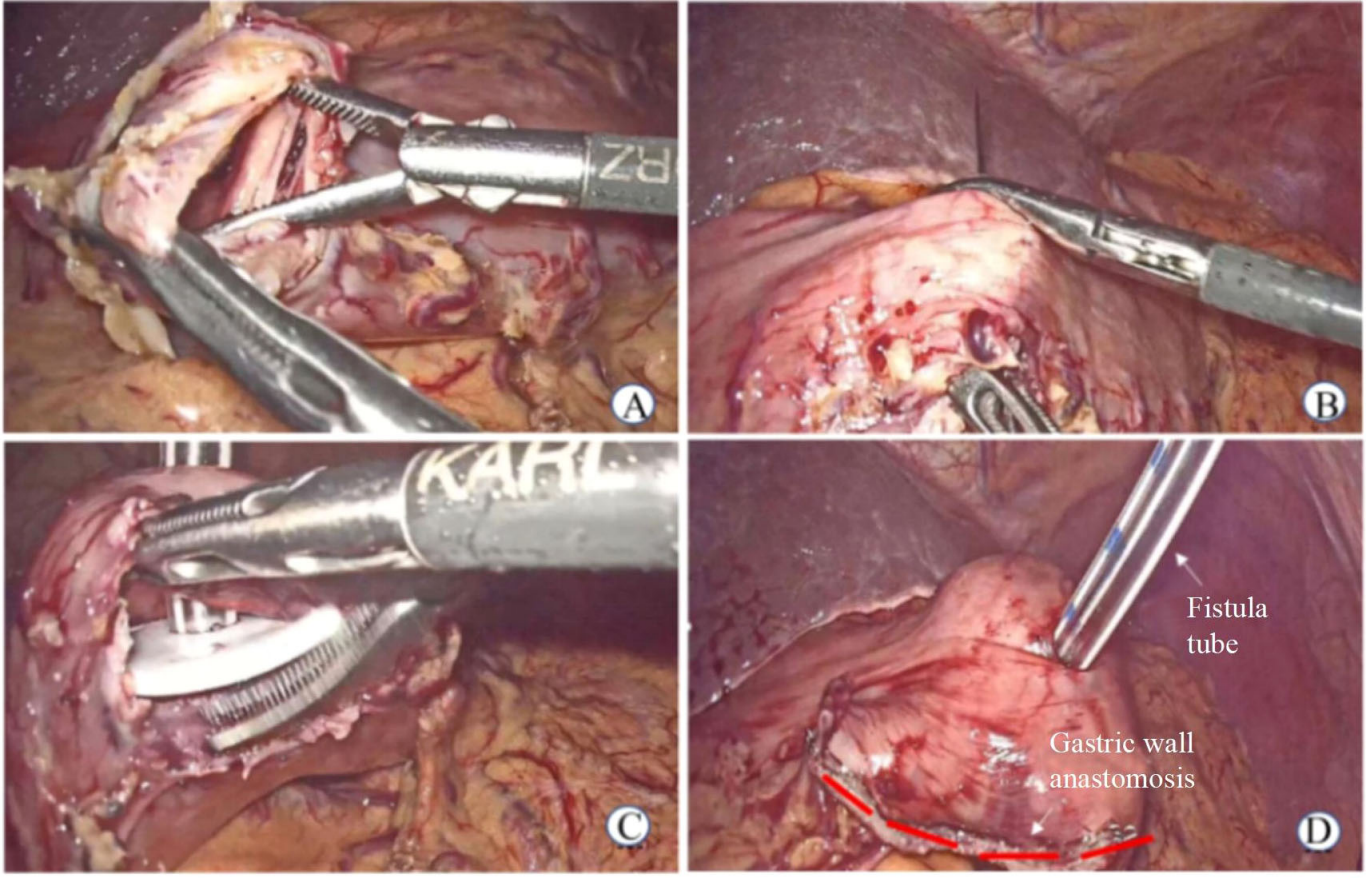
Laparoscopic gastrostomy. **(A)** Cut open gastric wall **(B)** The purse-string needle was inserted into the anterior wall of the stomach **(C)** The gastrostomy tube was placed **(D)** Postoperative status.

**Figure 3:**
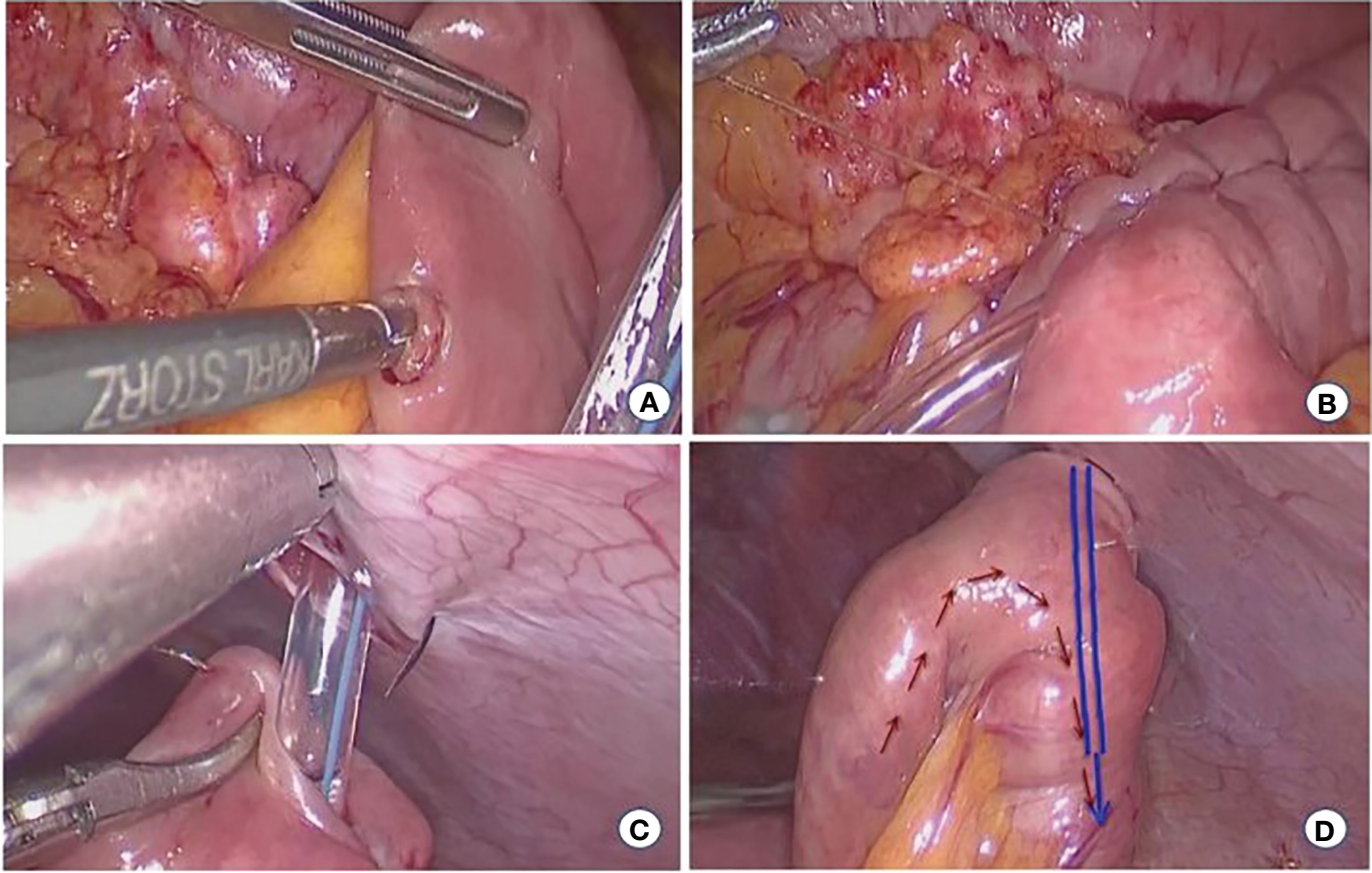
Laparoscopic jejunostomy. **(A)** Jejunum puncture **(B)** Embedding suture **(C)** Attach the fistula to the abdominal wall **(D)** Postoperative status.

(1) LGJ surgery was performed in patients with gastric pyloric cancer: a) The omentum of the greater curvature of the stomach was dissociated. We made holes in the greater curvature of the stomach (> 5 cm from the tumor edge) and jejunum (approximately 15 cm from the ligament of Treitz). b) The linear stapler was placed in the greater curvature of the stomach and closed with the jejunum to form a gastrojejunal anastomosis (**Figure 1**).

(2) Laparoscopic gastrostomy (LGT) was performed in patients with gastric cardia cancer: a) The omentum of the greater curvature of the stomach was dissociated, and the gastric wall was cut to 2 cm. b) The purse-string needle was inserted into the anterior wall of the stomach (2 cm from the incision of the gastric wall) and the abdominal wall from the inside, and the gastrostomy tube was placed by traction. c) The gastric wall incision was closed using a linear stapler, and the inner and outer latches of the fistula tube were fixed to the abdominal wall (**Figure 2**).

(3) Laparoscopic jejunostomy (LJT) was performed in patients with diffuse-type cancer. a) The jejunum was cut, using a linear stapler, 10–15 cm from the ligament of Treitz. b) The mesentery of the proximal jejunum was fully dissociated, and holes were made on the side of the proximal jejunum and the side of the jejunum 10 cm from the distal jejunum. A linear stapler was placed to perform side-to-side anastomosis of the proximal and distal jejunums. c) The distal jejunal stump was cut 2 cm, a purse-string needle was inserted into the intestinal wall (2 cm from the jejunal stump incision) and the abdominal wall from the inside, and the jejunostomy tube was placed. d)The opening of the distal jejunal stump was closed using a linear stapler, and the inner and outer latches of the fistula tube were fixed to the abdominal wall (**Figure 3**).

### 2.3 Conversion therapy

All patients received the EOX (epirubicin + oxaliplatin + capecitabine) regimen, and the EOX regimen was administered to patients who underwent LDTNR, 7–14 days post surgery. The EOX regimen was epirubicin 100 mg/m^2^ on day 1, oxaliplatin 130 mg/m^2^ on day 1, and capecitabine 825 mg/m^2^ on days 1-14, and the chemotherapy cycles repeated every 3 weeks. Imaging evaluations were performed after every two cycles of chemotherapy. After a multidisciplinary discussion, the tumor was evaluated for radical surgery. In the case of tumor progression or National Cancer Institute Common Terminology Criteria for Adverse Events (NCI-CTCAE) (8) grade 3-4 chemotherapy adverse events, subsequent treatments were formulated according to the multidisciplinary discussion.

### 2.4 Data collection

(1) Nutritional Risk Assessment: The total score of the Nutrition Risk Screening 2002 (NRS2002) was calculated (9). For nutritional status assessment, BMI and the Patient-Generated Subjective Global Assessment (PG-SGA) overall assessment grading were used (10). The Prognosis Nutrition Index (PNI) and Neutrophil-Lymphocyte ratio (NLR) were used to assess the inflammatory and immune status. We divided the patients into two groups: PNI (<45 vs. ≥45) and NLR (<2.5 vs. ≥2.5) (11, 12). The Quality of life Assessment: Spitzer QOL Index was used to evaluate patients’ mobility, daily life, health status, support, and knowledge of disease and life (13, 14). Nutritional risk, nutritional status, inflammatory and immune status, and quality of life were evaluated before surgery and 7 days and 1 month after surgery of patients in LDTNR group.
(2) Response to chemotherapy: The efficacy of chemotherapy was evaluated according to RECIST 1.1 criteria. The overall response rate (ORR) was calculated as follows: cases of complete response + cases of partial response)/total number ×100%, and the disease control rate (DCR) as follows: cases of complete response + cases of partial response + number of stable disease)/total number ×100%.
(3) Postoperative follow-up: Postoperative complications were graded according to the Clavien-Dindo classification, and patients were followed up by telephone, outpatient examination, and inpatient review until the death of the patient or the last follow-up date (November 1, 2021). OS was defined as the time from the start of treatment to the date of the last follow-up or death.

### 2.5 Statistics analysis

The SPSS Statistics 23.0 (IBM, USA) statistical software was used for statistical analysis. Continuous variables were presented as x ± s. Kruskal-Wallis H test, t-test, or Wilcoxon rank sum test was used for continuous variables. Categorical variables were described as absolute numbers or percentages and tested using the chi-square or Fisher’s exact test. The Kaplan-Meier method was used to analyze survival, and the log-rank test was used for survival analysis. Prognostic factors of OS were analyzed using univariate and multivariate logistic regression. A *P*-value of less than 0.05 was considered to indicate a statistically significant difference.

## 3 Results

### 3.1 Baseline characteristics

During the study period, we obtain data of 37 patients who received LDTNR therapy, and 33 patients who received chemotherapy only. The baseline characteristics of all eligible patients are outlined in **Table 1**. No significant differences were detected in sex, age, cancer types, ECOG, clinical stage, non-curable factors and baseline nutritional and inflammatory status. Baseline GOOSS was better in Non-LDTNR group in comparison to the LDTNR group (P<0.001).

**Table 1:** Baseline data for all patients.

		LDTNR (n=37)	Non-LDTNR (n=33)	P value
Sex				0.729
	Male	25 (67.6%)	21 (63.6%)	
	Female	12 (32.4%)	12 (36.4%)	
Age		62.95±12.09	58.9±9.49	0.127
Cancer types				0.766
	Gastric cardia cancer	5 (13.5%)	3 (9.1%)	
	Gastric pyloric cancer	24 (64.9%)	21 (63.6%)	
	Diffuse type	8 (21.6%)	9 (27.3%)	
ECOG				0.069
	0	0 (0)	2 (6.1%)	
	1	21 (56.8%)	11 (33.3%)	
	2	16 (43.2%)	20 (60.6%)	
GOOSS				<0.001
	0	12 (32.4%)	0 (0)	
	1	25 (67.6%)	0 (0)	
	2	0 (0)	33 (100%)	
cT				0.690
	T3	5 (13.5%)	2 (6.1%)	
	T4a	29 (78.4%)	27 (81.8%)	
	T4b	3 (8.1%)	4 (12.1%)	
cN				–
	0	0 (0)	0(0)	
	+	37 (100%)	33 (100%)	
Non-curable factor				
	Transverse colon invasion	1 (2.7%)	1 (3.0%)	0.935
	Pancreas invasion	2 (5.4%)	3 (9.1%)	0.550
	Liver metastasis	7 (18.9%)	8 (24.2%)	0.588
	Peritoneum metastasis	18 (48.6%)	17 (51.5%)	0.811
	No.16 lymph node metastasis	9 (24.3%)	4 (12.1%)	0.190
Borrman				0.747
	I	1 (2.7%)	1 (3.0%)	
	II	2 (5.4%)	4 (12.1%)	
	III	26 (70.3%)	20 (60.6%)	
	IV	8 (21.6%)	8 (24.2%)	
LDTNR				–
	LGT	5 (13.5%)	–	
	LGJ	24 (64.9%)	–	
	LJT	8 (21.6%)	–	
PNI				0.778
	<45	28 (75.7%)	24 (72.7%)	
	≥45	9 (24.3%)	9 (27.3%)	
NLR				0.574
	<2.5	8 (21.6%)	10 (30.3%)	
	≥2.5	29 (78.4%)	23 (69.7%)	
BMI		21.64±2.44	22.11±1.75	0.099

ECOG, Eastern cooperative oncology group; GOOSS, gastric outlet obstruction; LDTNR, Laparoscopic digestive tract nutrition reconstruction, LGT, Laparoscopic gastrostomy; LGJ, Laparoscopic gastrojejunostomy; LJT, Laparoscopic jejunostomy; PNI, Prognosis Nutrition Index; NLR, Neutrophil-Lymphocyte ratio; BMI, Body mass index.

### 3.2 Nutritional risk, nutritional status, inflammatory immune status, and quality of life

After LDTNR, the proportion of patients with nutritional risk (94.6% vs. 70.3% vs. 18.9%) and severe malnutrition (89.2% vs. 51.4% vs. 0) decreased gradually. The proportion of patients with NLR <2.5 (21.6% vs. 48.6% vs. 70.3%), PNI ≥45 (24.3% vs. 56.8% vs. 73.0%), and Spitzer QOL Index (P<0.05) increased gradually (**Tables 2**, **3**). There was no significant difference in the BMI (P>0.05) at these time points.

**Table 2:** The nutritional risk, inflammatory and immune status before operation, 7 days and 1 month after operation.

	NRS2002		PG-SGA			NLR		PNI		BMI
	Nutritional risk	Nutritional normal	A	B	C	<2.5	≥2.5	<45	≥45	
Before operation	35 (94.6%)	2 (5.4%)	0	4 (10.8%)	33 (89.2%)	8(21.6%)	29(78.4%)	28(75.7%)	9(24.3%)	21.64±2.44
7 days after operation	26 (70.3%)	11 (29.7%)	0	18(48.6%)	19 (51.4%)	18(48.6%)	19(51.4%)	16(43.2%)	21(56.8%)	21.39±2.31
1 month after operation	7 (18.9%)	30 (81.1%)	15(40.5%)	22(59.5%)	0	26(70.3%)	11(29.7%)	10(27.0%)	27(73.0%)	21.21±2.43
χ2/H	41.120		45.878			17.656		18.175		0.839
P	0.000		0.000			0.000		0.000		0.657

NRS2002, Nutrition Risk Screening; PG-SGA, Patient-Generated Subjective Global Assessment; NLR, Neutrophil-Lymphocyte ratio; PNI, Prognosis Nutrition Index; BMI, Body mass index.

**Table 3:** QOL scores at different periods.

Spitzer	QOL scores at different periods		
	Before operation	7 days after operation	1 month after operation
Total points	5.84±1.24	7.51±1.20	8.70±0.87
Activity	0.78±0.62	1.43±0.50	1.73±0.44
Daily Living	1.19±0.39	1.57±0.50	1.84±0.37
Health	1.00±0.33	1.30±0.46	1.73±0.44
Support	1.49±0.50	1.59±0.49	1.62±0.48
Outlook	1.35±0.48	1.62±0.48	1.78±0.41

QOL, Quality of life.

### 3.3 Response to chemotherapy

The median chemotherapy cycle of patients in LDTNR group was 6 cycles (2-10 cycles), higher than that in Non-LDTNR group (P<0.001). Among those who received LDTNR therapy, 2 patients had a complete response, 17 had a partial response, 8 had stable disease, and 10 had progressive disease, which was significantly better than the response rate in Non-LDTNR group(P<0.001) (**Table 4**). In the LDTNR group patients, peritoneal metastasis (receiving hyperthermic intraperitoneal chemotherapy) turned negative in eight patients, the No.16 lymph nodes disappeared or decreased in four patients, the depth of tumor invasion reduced to T4a stage in one patient with pancreatic invasion, and the liver metastasis significantly reduced or disappeared in three patients. While none of the Non-LDTNR group patients had resolution of non-curable factors.

**Table 4:** Response to chemotherapy.

		LDTNR (n=37)	Non-LDTNR (n=33)	P value
Median chemotherapy cycles		6 (2~10)	3 (2~6)	<0.001
Chemotherapy response				<0.001
	CR	2 (5.4%)	0 (0)	
	PR	17 (45.9%)	0 (0)	
	SD	8 (21.6%)	23 (69.7%)	
	PD	10 (27.0%)	10 (30.3%)	
	ORR	19 (51.4%)	0 (0)	
	DCR	27 (73.0%)	23 (69.7%)	
Subsequent gastrectomy		16 (43.2%)	0 (0)	<0.001

CR, complete response; PR, partial response; SD stable disease; PD, progression disease; ORR overall response rate; DCR, disease control rate

### 3.4 Surgical and postoperative situations

**Figure 4:**
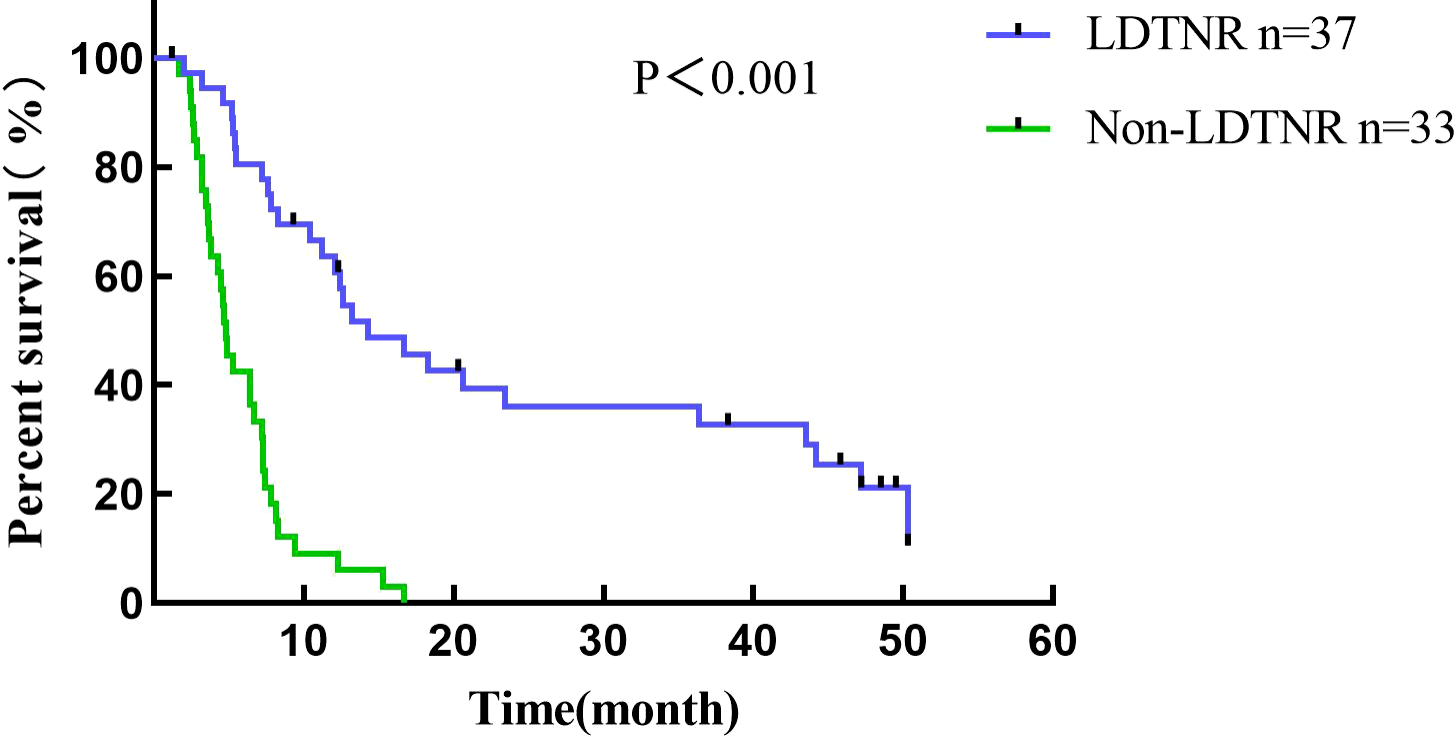
Survival curve of all patients enrolled in the present study. The 1-year cumulative survival rates of the patients with or without LDTNR were 59.5% and 9.1%. The 3-year cumulative survival rate with or without LDTNR was 29.7% and 0%, respectively.

**Figure 5:**
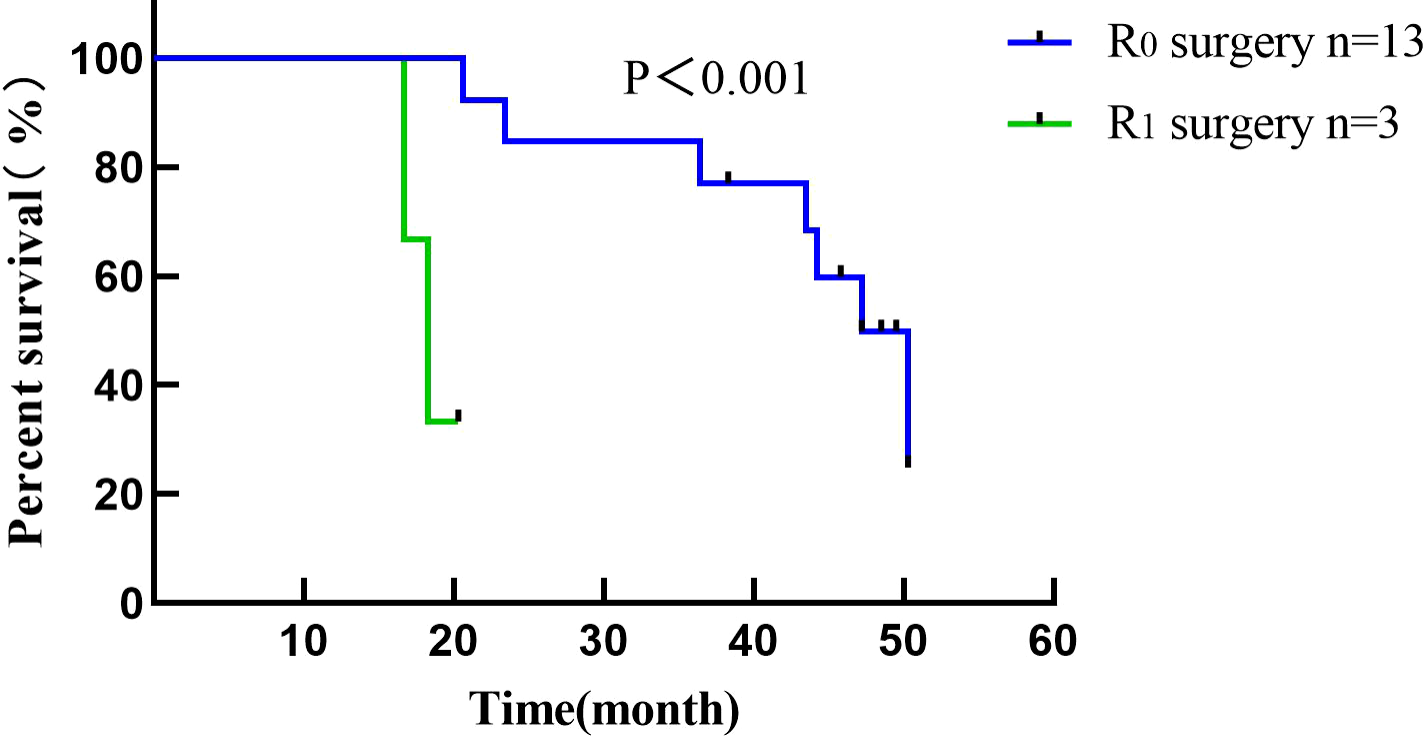
Survival curve of patients receiving radical gastrectomy in the present study. The survival time of patients with R0 resection was 41.9 ± 9.4 months and that of patients with R1 resection was 18.4 ± 1.5 months.

Conversion surgery was performed in 16 (43.2%) LDTNR group patients. Four patients underwent gastrectomy and D3 lymph node dissection, and 12 underwent gastrectomy and D2 lymph node dissection (including 3 patients with liver metastasis resection). R0 resection was achieved in 13 patients (81.2%) and R1 resection in 3 patients (18.8%) (**Table 5**). One patient (6.3%) developed grade III anastomotic leakage and was discharged after the endoscopic intervention. All patients were followed for 1.2-50.3 months, with a median time of 12.5 months. The 1-year cumulative survival rates of the patients with or without LDTNR were 59.5% and 9.1%. The 3-year cumulative survival rate with or without LDTNR was 29.7% and 0%, respectively. The difference in survival between the two groups was statistically significant (P<0.001) (**Figure 4**). Further analysis showed that the survival time of the 13 patients with R0 resection was 41.9 ± 9.4 months and that of the 3 patients with R1 resection 18.4 ± 1.5 months, and the difference was statistically significant (P<0.001) (**Figure 5**). Univariate and multivariate analysis identified LDTNR therapy and subsequent gastrectomy were associated with better long-term prognosis (**Table 6**).

**Table 5:** Postoperative pathological and complication outcomes.

		Conversion surgery(n=16)
Radical surgery		
	Laparoscopic total gastrectomy	7 (43.8%)
	Laparoscopic distal gastrectomy	9 (56.2%)
Resection margin		
	R0	13 (81.2%)
	R1	3 (18.8%)
pT		
	0	2 (12.5%)
	3	5 (31.3%)
	4a	9 (56.2%)
pN		
	0	11 (68.7%)
	1	5 (31.3%)
pM		
	0	0 (0)
Complication		
	Grade III	
	Leakage	1 (6.3%)

**Table 6:** Univariate and multivariate analyses for OS.

Variable	Hazard ratio	95% CI	P value
Univariate analysis			
Age (≥65/<65)	1.430	0.840-2.434	0.188
Sex (male/female)	1.128	0.659-1.932	0.661
ECOG (2/0 or 1)	1.430	0.840-2.434	0.188
BMI (<18.5/≥18.5)	1.111	0.443-2.790	0.822
PNI (<45/≥45)	0.849	0.457-1.578	0.605
NLR (<2.5/≥2.5)	0.922	0.524-1.622	0.778
Subsequent gastrectomy (yes/no)	53.805	9.311-310.926	<0.001
Treatment selection			
LDTNR	Ref	Ref	Ref
Non-LDTNR	5.198	2.845-9.496	<0.001
Multivariate analysis			
Age (≥65/<65)	1.140	0.655-1.985	0.642
ECOG (2/0 or 1)	0.721	0.408-1.274	0.260
Subsequent resection (yes/no)	51.296	6.399-411.205	<0.001
Treatment selection			
LDTNR	Ref	Ref	Ref

ECOG, Eastern cooperative oncology group; BMI, Body mass index; LDTNR, Laparoscopic digestive tract nutrition reconstruction; PNI, Prognosis Nutrition Index; NLR, Neutrophil-Lymphocyte ratio; BMI, Body mass index.

## 4 Discussion

In previous studies, palliative resection was often used for patients with unresectable advanced gastric cancer with obstructive symptoms, focusing less on the long-term prognosis of such patients (14). A previous study (15) showed that tumor cells are released into the blood after palliative resection, and the activity of residual tumor cells is enhanced under surgical stress and an inflammatory response, which greatly enhances proliferation, invasion, and metastasis. In addition, the influence of gastrectomy on the quality of life, chemotherapy compliance, and tolerance will also affect the subsequent treatment effects in patients with gastric cancer. The REGATTA study (14) demonstrated that palliative resection could not prolong patient survival. LDTNR can bypass obstruction without stimulating the primary tumor, relieve the symptoms caused by obstruction, and restore enteral nutrition. It is expected to overcome the problem of undertaking subsequent treatments in patients with obstruction due to long-term insufficient nutrient intake and a metabolic state of high decomposition and low synthesis of nutrients (14, 16, 17). To the best of our knowledge, this study is the first to analyze the role of LDTNR in improving the inflammatory nutritional immune status and survival time of patients with unresectable advanced gastric cancer with obstructive symptoms.

In patients with gastric cancer obstruction, improvement in nutritional status enhances long-term survival. Therefore, relieving obstruction plays an important role. LDTNR is associated with less trauma load, shorter recovery time for enteral nutrition, and high compliance of patients with subsequent treatment (6). While reducing the stress response, it also avoids failure of endoscopic catheterization caused by obstruction and gastric retention, stent displacement, and secondary interventions due to tumor progression (18). After LDTNR, the first time to liquid food was after 1.43 ± 0.50 days, which was shorter than that after an open procedure (19), indicating that LDTNR results in less trauma and faster recovery of intestinal function. In addition, the latest general principles of nutritional therapy indicate that it supplements insufficient nutrients, enhances the body’s immune function, and reduces the inflammatory response (20). After LDTNR, the nutritional status and quality of life gradually increased, the inflammatory indicators gradually decreased, and the differences were statistically significant (P<0.001).

In survival analysis, LDTNR therapy offers a survival benefit in patients with unresectable and obstructive gastric cancer. The 3-year cumulative survival rate of patients with LDTNR was 29.7%, which is higher than that in Non-LDTNR group (P<0.001). In addition, multivariate analysis identified that LDTNR were associated with better long-term survival, compared with Non-LDTNR (HR, 5.198; 95% CI 1.019-3.368,P<0.001). For patients with GO caused by unresectable advanced gastric cancer, subsequent gastric resection, specifically R0 resection, is key to determining long-term prognosis. In this study, the survival of patients treated with radical resection was significantly better than those without this treatment. The median survival time of patients with R0 resection was also significantly longer than that of patients with R1 resection, which was similar to the previous report (21). Such patients have a low completion rate of conversion therapy owing to their poor nutritional status. In this study, 37 patients with unresectable advanced gastric cancer received LDTNR; the ORR was higher than that in Non-LDTNR group, with a resection rate of 43.2% and an R0 resection rate of 81.2%, which are also higher than those previously reported (22). The basic nutritional status of patients with gastric cancer and obstruction is poor. Preoperative chemotherapy often leads to a decreased nutritional status and suppression of cellular immunity, which increases the risk of postoperative complications. In this study, only one patient who underwent LDTNR had grade III postoperative complications, which was lower than that reported in the literature (20), and no deaths occurred. Enteral nutrition can effectively reduce gastric wall edema and malnutrition caused by obstruction and reduce the occurrence of anastomotic leakage, bleeding, and other complications. However, there was no significant difference in BMI among the patients in this study, and 10 patients could not tolerate the toxic effects of chemotherapy. This suggests that after the removal of the obstruction, individualized enteral nutrition, appropriate loading exercise, and efficacy evaluation are still needed for the safety and effectiveness of conversion therapy.

## 5 Conclusion

LDTNR results in better nutritional and inflammatory immune status of patients with obstruction caused by gastric cancer and improves the long-term prognosis of these patients. However, the effect of combination therapy with conversion therapy on the efficacy of chemotherapy, postoperative complications, and OS of these patients still needs to be verified using larger sample size and long-term follow-up data.

## Data availability statement

The raw data supporting the conclusions of this article will be made available by the authors, without undue reservation.

## Ethics statement

The studies involving human participants were reviewed and approved by Ethics Committee of the Fujian Provincial Hospital. The patients/participants provided their written informed consent to participate in this study.

## Author contributions

Conceptualization, BH and GG. Methodology, RY and CW. Formal analysis, RY and CW. Resources, RY and CW. Writing—original draft preparation, RY and CW. Writing—review and editing, RY, CW, BH and GG. Funding acquisition, GG. All authors contributed to the article and approved the submitted version.
